# AGuIX nanoparticles as a promising platform for image-guided radiation therapy

**DOI:** 10.1186/s12645-015-0012-3

**Published:** 2015-09-02

**Authors:** Alexandre Detappe, Sijumon Kunjachan, Joerg Rottmann, James Robar, Panagiotis Tsiamas, Houari Korideck, Olivier Tillement, Ross Berbeco

**Affiliations:** Radiation Oncology Department, Dana-Farber Cancer Institute, Brigham and Women’s Hospital, Harvard Medical School, Boston, MA 02215 USA; Institut Lumière-Matière, Université Claude Bernard, 69000 Lyon, France; Department of Medical Physics, Nova Scotia Cancer Centre, Dalhousie University, Halifax, NS B3H 1V7 Canada

**Keywords:** Nanomedicine, MRI, Gadolinium, Dose enhancement, Flattening filter free

## Abstract

AGuIX are gadolinium-based nanoparticles developed mainly for imaging due to their MR contrast properties. They also have a potential role in radiation therapy as a radiosensitizer. We used MRI to quantify the uptake of AGuIX in pancreatic cancer cells, and TEM for intracellular localization. We measured the radiosensitization of a pancreatic cancer cell line in a low-energy (220 kVp) beam, a standard 6 MV beam (STD) and a flattening filter free 6 MV beam (FFF). We demonstrated that the presence of nanoparticles significantly decreases cell survival when combined with an X-ray beam with a large proportion of low-energy photons (close to the k-edge of the nanoparticles). The concentration of nanoparticles in the cell achieves its highest level after 15 min and then reaches a plateau. The accumulated nanoparticles are mainly localized in the cytoplasm, inside vesicles. We found that the 6 MV FFF beams offer the best trade-off between penetration depth and proportion of low-energy photons. At 10 cm depth, we measured a DEF_20 %_ of 1.30 ± 0.47 for the 6 MV FFF beam, compared to 1.23 ± 0.26 for the 6 MV STD beam. Additional measurements with un-incubated nanoparticles provide evidence that chemical processes might also be contributing to the dose enhancement effect.

## Background

Nanoparticles made from high-Z materials are promising agents to increase radiosensitivity of cancer cells during the application of radiation therapy. Hainfeld et al. (2004, 2008) demonstrated therapeutic enhancement with gold nanoparticles (GNP) in a 250 kVp X-ray beam. The dose-enhancing effect was attributed to the photoelectric effect and the increased generation of Auger electrons (Pradhan et al. 2009; Jelveh and Chithrani 2011; Dorsey et al. 2013; Kumar et al. 2013). Further studies have confirmed the dose-enhancing effect of GNP in 6 MV X-ray beams, an energy range that is typically used for clinical radiation therapy (Detappe et al. 2013; Berbeco et al. 2011; Cho 2005; Jones et al. 2010; Lin et al. 2014; Robar et al. 2002; McMahon et al. 2008; Ngwa et al. 2014).

However, even though gold nanoparticles may be efficient radiosensitizers, it remains difficult to measure their exact concentration within the tumor with current clinically available imaging methods. To address this issue, multimodal nanoparticles have been proposed that combine both therapeutic and diagnostic functionality. Mignot et al. 2013 developed a gadolinium-based nanoparticle, AGuIX, which is a non-toxic magnetic resonance contrast agent and sufficiently small (sub-5 nm diameter) to allow for renal clearance (Le Duc et al. 2014). With an atomic number of *Z* = 64, gadolinium is a high-Z material and therefore also contributes substantial radiation dose enhancement. (Sancey et al. 2014).

Previous assessment of AGuIX nanoparticles has mostly focused on their imaging properties (Di Corato et al. 2013; Le Duc et al. 2011; Bianchi et al. 2014a, b; Paul et al. 2014). In vitro experiments have included different cell lines and preclinical irradiation beams (microbeam radiation therapy, low-energy X-ray). Researchers have shown early evidence of radiosensitization (Bianchi et al. 2013; Aspord et al. 2013; Mowat et al. 2011; Porcel et al. 2014; Stefančíková et al. 2014; Luchette et al. 2014). In addition, in vivo experiments with intravenous or intra-tumoral injection for subcutaneous and orthotopic lesions have been performed in preclinical radiation beams (Le Duc et al. 2011; Bianchi et al. 2014a).

The aim of the current study is to characterize the uptake of the AGuIX nanoparticles in pancreatic cancer cells by magnetic resonance imaging (MRI), and investigate the radiation dose enhancement effect attributable to AGuIX when irradiating the cells with a clinical linear accelerator at 6 MV. This represents the first step towards demonstrating an in vivo effect for pancreatic cancer. The AGuIX platform is particularly relevant for pancreatic cancer due to the poor prognosis, proximity of organs at risk, and targeting difficulty for this disease in radiation therapy. AGuIX offers a solution that is highly compatible with the current trend in radiation oncology towards MRI-based patient simulation and MRI-Linac treatment devices (Raaymakers et al. 2011; Keall et al. 2014). There may be a strong future for AGuIX in future MRI-guided radiation therapy for pancreatic cancer.

## Methods

### Cell culture

Panc1 cells were cultured in Dulbecco’s Modified Eagle Medium (DMEM), supplemented with 10 % fetal bovine serum (FBS) (Sigma, USA), 1 % Penicillin Streptomycin Glutamine (Invitrogen, USA) and were stored in a humidified incubator at 37 °C and 5 % CO_2_.

### AGuIX nanoparticles (Nano-H Inc., France)

**Fig. 1:**
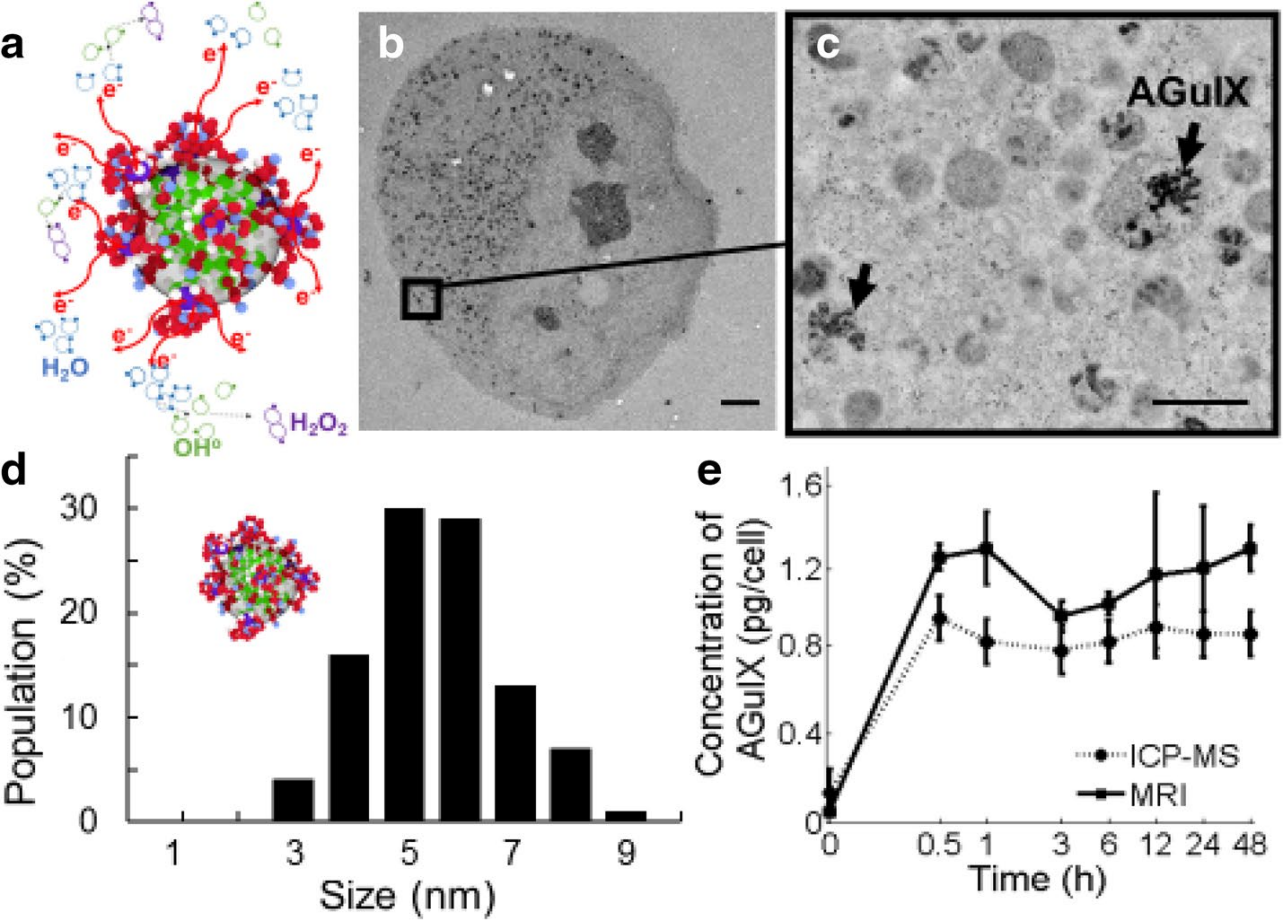
AGuIX uptake studies. **a** Schematic representation of the activation induced by the external X-ray irradiation on the AGuIX. **b** TEM images (3000×) depict the active endocytosis uptake of AGuIX into the tumor cells. *Bar* 5 µm. **c** Magnified TEM image (25000×) shows AGuIX nanoparticles captured by endosomal vesicles (*black arrow*) and carried into the cytoplasm. *Bar* 1 µm. **d** Hydrodynamic measurement of the AGuIX size. **e** Panc1 cells were incubated for different time points with 0.5 mM of AGuIX. The concentration in pg/cell measured by MRI and crosschecked by ICP-MS. The MRI measurement is determined with a calibration curve allowing the translation between the concentration in AGuIX and the relaxivity time. For both methods of measurement, the uptake plateaus after 30 min

The nanoparticles are composed of a polysiloxane shell surrounded by DOTA (1,4,7,10-tetra-azacyclododecane-1-glutaric anhydride-4,7,10-triacetic acid) covalently bound to the inorganic matrix and Gadolinium (Fig. 1a). The size of each nanoparticle was measured by dynamic light scattering (DLS) and is 5 ± 0.1 nm (Fig. 1d) with a mass of 10 ± 1 kDa. More detailed information on the production process of the nanoparticles may be found in the references (Detappe et al. 2013).

### AGuIX uptake analysis with MRI and ICP-MS

The concentration of the nanoparticles inside the cells was analyzed with a Bruker Biospin 7T Magnetic Resonance Imaging (MRI) scanner. First, a calibration curve without cells was acquired for various concentrations of nanoparticles in the cell culture solution. The concentration of AGuIX particles used for the experiment is given in gadolinium equivalent species and chosen based on published literature (Rima et al. 2013). Cells were incubated with 0.5 mM (0.5 mg/L) of AGuIX at 37 °C and 5 % CO_2_ for 30 min, 1 h, 3 h, 6 h, 24 h and 48 h. After incubation, the cells were washed and trypsinized to remove any excess nanoparticles in the solution before scanning. All MRI scans featured a RARE-T1 map imaging-sequence with 2 mm slice thickness, repetition time of 10 ms, echo time of 21.4 ms, echo train length of 4, flip angle of 180°, and matrix size 256 × 128 pixels. Each measurement was performed in triplicate.

Inductively coupled plasma mass spectrometry (ICP-MS) was used to validate the MRI results. For this procedure, the cells were dissolved in a radio-immuno precipitation RIPA buffer and then suspended in water until analysis. ICP-MS is used to determine the exact quantity of Gadolinium. As with the MRI measurements, incubation times were 30 min, 1 h, 3 h, 6 h, 24 h and 48 h. Each time point was measured in triplicate.

### Localization of AGuIX within Panc1 cells

Transmission electronic microscopy (TEM) was performed to investigate the local nanoparticle distribution within the cells. A concentration of 0.5 mM of AGuIX was incubated for 1 h with Panc1 cells, followed by washing out residual nanoparticles and staining with 4 % formaldehyde and 1 % glutaraldehyde in 0.1 M Pb for imaging.

### Irradiation and setup

We investigated cell response to low-energy and high-energy photon irradiation for a dose range of 2–10 Gy. For low-energy experiments, we used a Small Animal Radiation Research Platform (SARRP) operated at 220 kVp (0.15 mm Cu filter) and a source to cell distance of 35 cm. For high-energy irradiations, we used a clinical linear accelerator (TrueBeam, Varian Inc, USA) operated at 6 MV with and without a flattening filter in the beam line. Using a flattening filter is the current standard (STD) in radiation therapy while the use of flattening filter free beams (FFF) is still relatively novel to clinical application. Cells were placed on 5 cm of water equivalent material and another 10 cm on top to allow for full scattering and back scattering conditions. The source to cell distance was 100 cm, field size 10 × 10 cm^2^ and dose rate 400 cGy/min for both STD and FFF deliveries.

### Clonogenic assay

Panc1 cells were incubated in DMEM with 0.5 mM of AGuIX following the specifications below, and then irradiated. After irradiations, the cells were incubated for another 4 h; afterwards they were washed with PBS, trypsinized and counted. The cells were replated in 10 cm dishes at 300 cells per plate and allowed to grow for 10 days, before staining with a 1 % crystal violet and 10 % ethanol dye solution. The platting efficiency was 67 ± 7 %. The plates were digitally scanned and automatically counted with software developed in our group. Measurements were performed in triplicate.

For the 6 MV irradiations, the cells were incubated 1 h and the medium unwashed. For the low-energy photon experiments, 4 different configurations were investigated:

A. Irradiation without any nanoparticles.
B. Cells were not incubated with nanoparticles. Nanoparticles were placed in the media just prior the irradiation. We called this configuration +IR/−incubation.
C. Cells were incubated with the nanoparticles and the media was changed just prior the irradiation. We called this +IR/+washing.
D. Cells were incubated with the nanoparticles and were irradiated. We called this +IR/−washing.

The (B) experiment (+IR/−incubation) is a test of the hypothesis that a dose enhancement effect can be caused by nanoparticles located outside the cell. A positive result supports the presence of some long-range effects beyond just photoelectric and Auger processes.

### Data analysis

We quantified the effect of the nanoparticles utilizing three methods commonly found in the literature (Sancey et al. 2014; Jain et al. 2011; Chithrani et al. 2010; Roeske et al. 2007). The Dose Enhancement Factor (DEF) is the ratio of the area between the survival curves with and without nanoparticles. The DEF_20 %_ is the ratio of doses at 20 % survival for irradiation without nanoparticles versus with nanoparticles. The sensitivity enhancement ratio at 4 Gy (SER_4Gy_) is the ratio of survival fractions at 4 Gy for irradiation without and with nanoparticles.

To characterize the radiation response of the Panc1 cells with and without AGuIX, we employed the classical linear quadratic model (LQM):

$$S\left( D \right) \, = \, \exp \left( { - \, \alpha D \, - \, \beta D^{2} } \right)$$

Here, *S* is the cell survival fraction, *D* the irradiation dose and *α* and *β* are parameters representing direct lethal and sub-lethal damage, respectively. Statistical significance of cell survival changes with the application of nanoparticles is calculated using a Kruskal–Wallis test.

## Results and discussion

### Uptake measurement

Results from both MRI and ICP-MS uptake analyses are shown in Fig. 1. Both methods give consistent results and show that AGuIX uptake saturates at about 1.25 pg per cell after 30 min incubation time for 0.5 mM of AGuIX incubated.

TEM imaging (Fig. 1b, c) reveals that after 1 h of incubation, the nanoparticles are predominantly localized in vacuoles in the cytoplasm. This result is in agreement with the previously published studies that applied AGuIX to other cell lines (Stefančíková et al. 2014; Rima et al. 2013).

MRI and ICP-MS results both show a constant level of nanoparticles inside the cells after 30 min of incubation. The MRI measurement is systematically larger than the ICP-MS measurement. This is because the T1 signal is sensitive to the size of nanoparticles, which decrease during hydrolysis (which occurs due to the low AGuIX concentration) (Le Duc et al. 2014).

### Activation of the nanoparticles

**Fig. 2:**
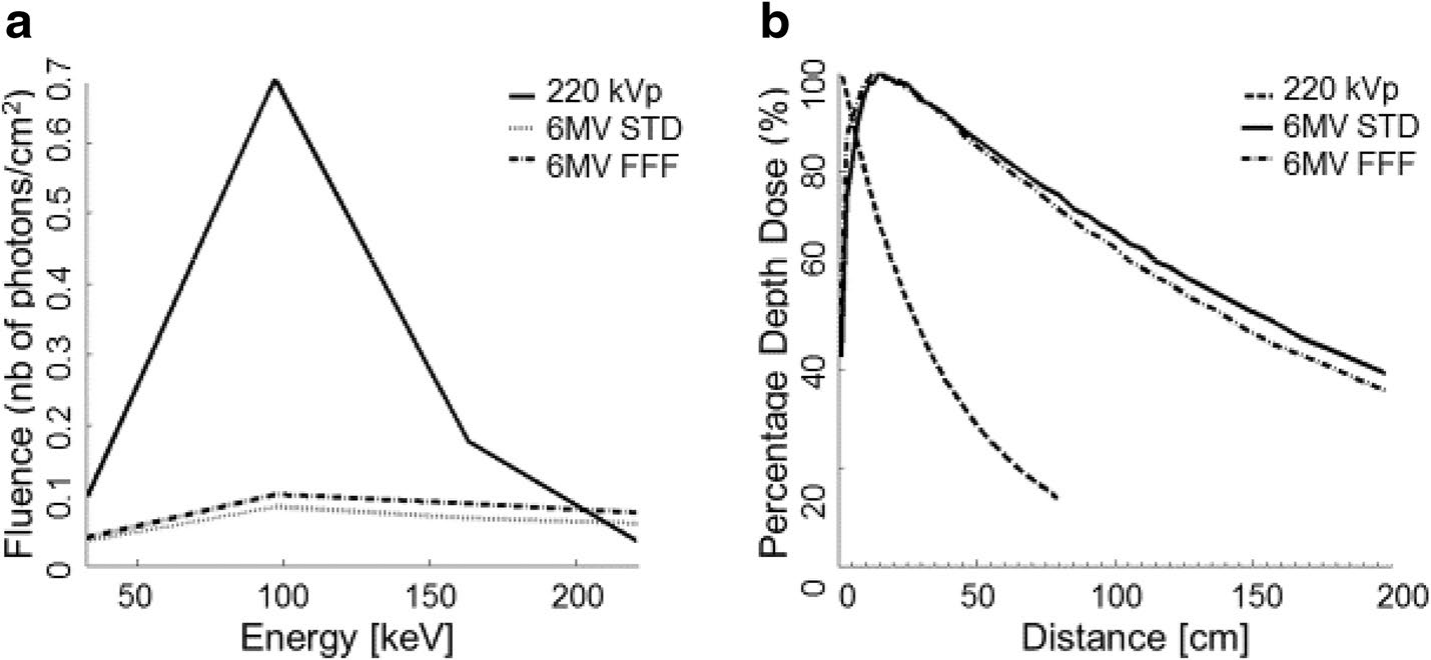
Representation of the difference between the preclinical beam (SARRP) and the clinical beam. **a** The 3 different spectra (220 kVp, 6 MV FFF, and 6 MV STD) were represented near the k-edge of the Gadolinium (50.2 kV). Calculation were performed with Monte Carlo simulation at 35 cm source–skin distance; 12 mm circular field size for the 220 kVp machine, and at 90 cm source–skin distance; 10 cm depth; 10 × 10 cm^2^ field size for the 6 MV irradiations. **b** Percentage depth dose (PDD) for the 3 photon beams. The maximum dose for the 220 kVp occurs at the surface while it is at approximately 1.5 cm for both 6MV beams

For the highest probability of a photoelectric interaction with the K-shell of Gadolinium, the incident photon should have an energy just above 50 keV. Figure 2a shows a comparison of the low-energy photon spectra for the beams used in this study. Spectra are generated using EGSnrc Monte Carlo code (Tsiamas et al. 2011, 2014).

From these distributions, it is clear that 220 kVp is expected to have the highest dose enhancement. However, it is not clinically feasible to use low-energy photons to treat the majority of patients (especially pancreatic cancer patient) due to the poor penetration depth and skin-sparing properties. Figure 2b shows a comparison of the percentage depth dose (PDD) for the radiation beams used in this study. The best trade-off between penetration depth and proportion of low-energy photons exists for the 6 MV FFF beam. Further modifications of the 6 MV beam, including the use of a low-Z target, (Parsons et al. 2014) may also improve the dose enhancement while preserving the deep penetration properties.

### Dose enhancement effects

**Fig. 3:**
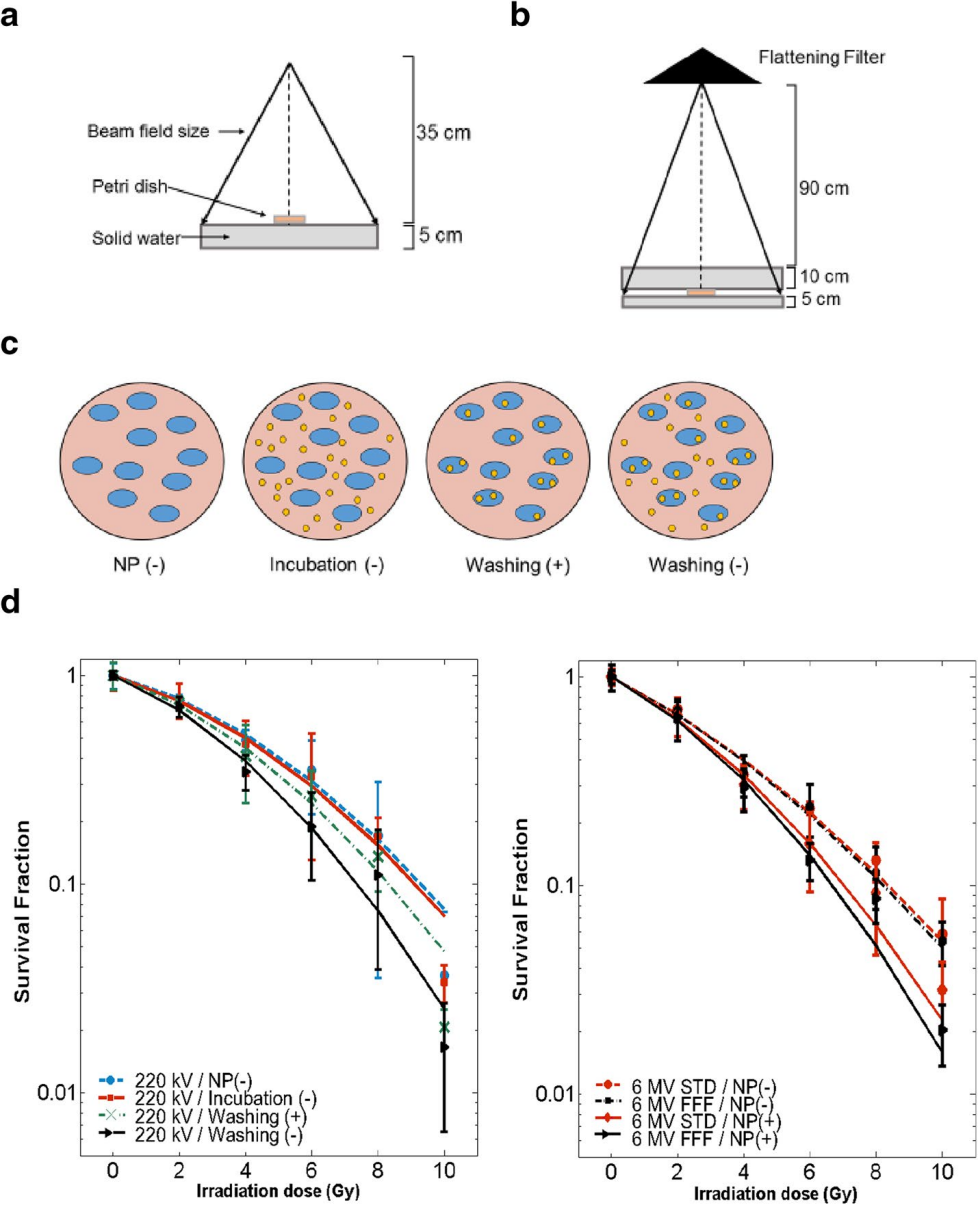
Radiation dose enhancement studies. Clonogenic assays of Panc1 tumor cells post-AGuIX incubation (1 h). **a** Schematic view of the irradiation setup for the preclinical beam (220 kVp) and **b** for the clinical beam (6 MV). The flattening filter is removed for the FFF irradiations. **c** Schematic representation of the clonogenic assay for the preclinical irradiations. Clinical irradiations were performed with the “washing (-)” situation. *Blue circles* represents the cells. *Yellow circles* represent the nanoparticles. **d** Preclinical irradiation setup (SARRP) was used to induce radiation enhancement effect in AGuIX using a 220 kVp beam (*left*) and clinical irradiation setup (6 MV beam) was used to treat cells with (STD) and without (FFF) flattening filter (*right*). Linear quadratic models were fitted to experimental data

A significant radiosensitization effect was observed for all the clonogenic assays performed (Fig. 3). Results of the dose enhancement studies are summarized in Table 1. The dose enhancement effect appears to increase with the proportion of low-energy photons in the spectrum as expected (Detappe et al. 2013; Berbeco et al. 2011). For the high-energy (clinical) photon beams, cell killing is significantly increased by the removal of the flattening filter in the FFF beam, compared to the STD beam (*p* = 0.014, Wilcoxon). The SER_4Gy_ is 1.20 ± 0.04 for the FFF irradiation and 1.12 ± 0.04 for the STD. The DEF_20 %_ is 1.30 ± 0.05 for the FFF and 1.23 ± 0.03 for the STD.

**Table 1:** Dose enhancement effect in terms of DEF, DEF_20 %_ and SER_4Gy_ for Panc1 cells incubated with 0.5 mM AGuIX

Irradiation					
Preparation	220 kVp			6 MV FFF	6 MV STD
	AGuIX no incubation	AGuIX 1h incubation washing	AGuIX 1 h incubation unwashing	AGuIX 1 h incubation unwashing	
DEF	1.09	1.17	1.46	1.23	1.19
DEF 20 %	1.01	1.1	1.31	1.3	1.23
Sensitivity (SER4Gy)	1.05	1.19	1.41	1.2	1.12
*p* value	0.131	0.038	***	0.009	0.011

*p* values were calculated using a Kruskal Wallis to test the effect of the nanoparticles with the control

*** *p* < 0.001

While the physical property of the incident radiation beams clearly provides a therapeutic advantage, there may be other factors contributing as well. Figure 1b, c shows the nanoparticles clustered inside the cytoplasm, away from the DNA. Radiosensitization was observed for this scenario as well as for non-incubated nanoparticles (located outside the cell) (Fig. 3d). McMahon et al. (2011) demonstrated the role of Auger electrons to create a local effect and the impact of nanoparticle clustering. This local effect increases the formation of reaction oxygen species (ROS), such as OH°, H_2_O_2_, or HOCl. Some of these ROS have high chemical stabilities and a long-range action (few mm) that may increase the cell death even if the nanoparticles are not localized in the cells. The results shown in Fig. 3d are similar to those obtained by Porcel et al. (2014) for hadrontherapy. Thus, in addition to the physical properties, a biological or chemical effect should be explored to explain the measured radiosensitization.

## Conclusion

AGuIX nanoparticles create significant dose enhancement in Panc1 cell lines for low- and high-energy photon irradiation. Using a 6 MV flattening filter free (FFF) beam improves dose enhancement compared to a standard 6 MV beam. Because of their contrast in MRI images, AGuIX have excellent potential as theranostic agents (Lux et al. 2011; Kunjachan et al. 2012).
